# Reverse surface-polariton cherenkov radiation

**DOI:** 10.1038/srep30704

**Published:** 2016-08-01

**Authors:** Jin Tao, Qi Jie Wang, Jingjing Zhang, Yu Luo

**Affiliations:** 1 School of Electrical and Electronic Engineering, Nanyang Technological University, 639798, Singapore

## Abstract

The existence of reverse Cherenkov radiation for surface plasmons is demonstrated analytically. It is shown that in a metal-insulator-metal (MIM) waveguide, surface plasmon polaritons (SPPs) excited by an electron moving at a speed higher than the phase velocity of SPPs can generate Cherenkov radiation, which can be switched from forward to reverse direction by tuning the core thickness of the waveguide. Calculations are performed in both frequency and time domains, demonstrating that a radiation pattern with a backward-pointing radiation cone can be achieved at small waveguide core widths, with energy flow opposite to the wave vector of SPPs. Our study suggests the feasibility of generating and steering electron radiation in simple plasmonic systems, opening the gate for various applications such as velocity-selective particle detections.

When a charged particle travels at a velocity faster than the phase velocity of light in a medium, it can drive the medium to emit electromagnetic radiation with a forward pointing wavefront^1^. This unique phenomenon is called Cherenkov radiation, named after the Russian scientist Pavel Alekseyevich Cherenkov, the 1958 Nobel Laureate who was the first to observe it experimentally. Cherenkov radiation has been widely studied in conventional media, enabling a wide range of applications, including the measurement of fast particles in high-energy physics^2^,^3^, the detection of labeled biomolecules^4^,^5^, the characterization of fission rate in nuclear reactors^6^,^7^, and determining properties of high-energy astronomical objects^8^,^9^. In 1968, Veselago predicted that the cone of the radiation will be directed backward relative to the motion of particle in negative index media, featuring the reverse Cherenkov radiation^10^. Later theoretical studies suggests that backward propagating Cherenkov radiation behavior can be expected in photonic crystals within one particle-velocity range^11^. Facilitated by the metamaterial technology, reverse Cherenkov radiation has recently received intensive attentions^12^,^13^. Its unusual feature was experimentally verified in metamaterials at microwave frequencies^14^,^15^, where a waveguide with an array of open slots was used to mimic a moving charged particle. However, the direct experimental observation of reverse Cherenkov radiation is still rarely reported, as it is always challenging to fulfill the requirement that high speed electron beams propagate in negative index metamaterials for transversely magnetic polarized waves, at a speed *v* > |*v*_*p*_| (Here *v*_*p*_ is the phase velocity of electromagnetic waves in the metamaterial).

Recent studies have brought great attentions to Cherenkov radiation in plasmonic systems. SPPs are electromagnetic excitations that propagate along the interface between a metal and a dielectric medium, and can be excited by electrons or photons. Since the wave vector of SPPs is larger than that in the free space, it is possible to satisfy the Cherenkov radiation condition by properly designing the dispersion property of a plasmonic system. Theoretical work shows that in a structure of metallic film with loaded dielectric, the SPPs excited by electrons can be transformed into Cherenkov radiation^16^,^17^. In a recent experimental study, controlled steering of Cherenkov surface plasmon wakes has been realized with a one-dimensional metamaterial by changing the angle of incident and the photon spin angular momentum of the incident radiation^18^. Although the SPPs approach greatly lower the barrier for the observation of Cherenkov radiation, the reverse Cherenkov radiation for surface plasmons has not yet been discussed.

Here, we study analytically the reverse Cherenkov radiation for surface plasmons. By considering the dispersion relation for SPPs in a metal-insulator-metal (MIM) waveguide, the condition for generating Cherenkov radiation condition in this plasmonic system is obtained. In particular, we find that the Cherenkov radiation switches from forward to backward as the core thickness of the waveguide is reduced, where the dispersion curve of a transverse magnetic (TM) surface plasmon mode with a negative slope appears. Detailed derivations for radiated electric and magnetic fields are performed in both frequency and time domains, revealing a radiation pattern with a backward-pointing radiation cone at small waveguide core width, with energy flow opposite to the wave vector of SPPs. Our study introduces a new approach of realizing reverse Cherenkov radiation, without involving metamaterials which requires complex fabrication. A possible practical setup for experimental demonstration is suggested and verified with finite-difference time-domain (FDTD) simulations. Our work gives guidance for designing plasmonic system to realize different types of Cherenkov radiation, opening the gate for various applications such as velocity-selective particle detections.

## Results and Discussion

We start our discussion with an electron moving along the *z* direction with a constant velocity, generating an equivalent current given by^19^:


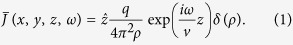


The electric and magnetic fields associated with the moving electron in free space take the following forms:


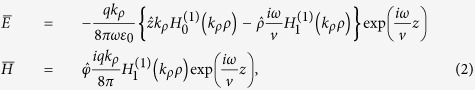


where *q* = −1.6 × 10^−19^ C is the charge taken by the electron, *v* is the velocity of the electron, 
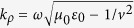
, and 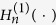 is the first kind Hankel function of the *n*-th order.

To facilitate the derivations for more complicated cases, we introduce a vector potential 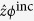 along the propagation *z* direction and rewrite Eq. (2) as:


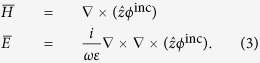


where 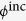 is a scalar potential, characterizing the amplitude of the vector potential Comparing Eq. (2) with Eq. (3), we find that 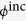 take the following form:


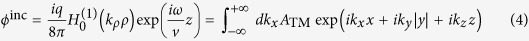


where we defined *k*_*z*_ = *ω*/*v* and 
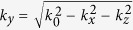
. The expansion coefficient *A* is calculated as


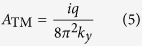


**Figure 1:**
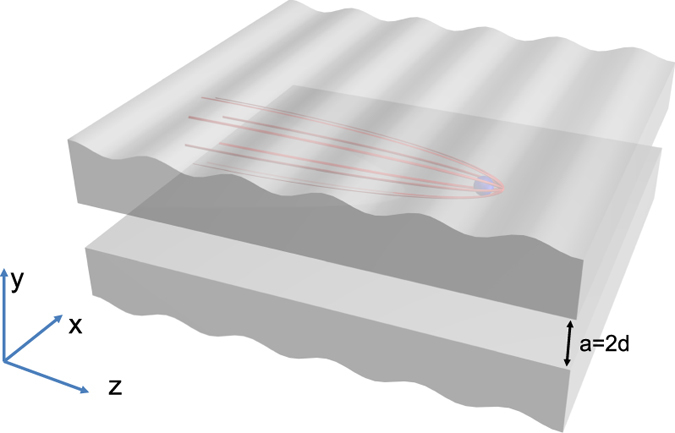
Schematic of an electron moving in a MIM waveguide structure.

We consider the electron moving inside a MIM waveguide (shown in Fig. 1), on the plane separated by *d* from each metal surface. Here, the metal is characterized by Drude model 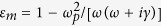, with plasma frequency *ω*_*p*_ = 8 eV and damping coefficient *γ* = 0.032 eV, and the insulator is assumed to be air. The fields can be decomposed into TE and TM components with respect to the *z* axis, characterized by vector potentials 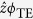 and 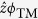, respectively. The electromagnetic fields can then be expressed as^20^,^21^:


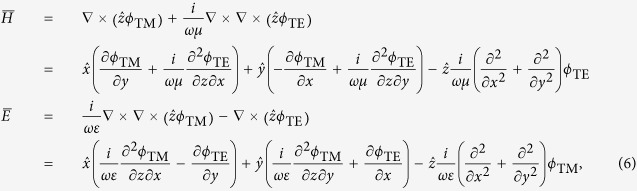


Comparing Eq. (6) with Eq. (2), we can see that the source fields only have the TM components. The potentials in each region can be listed as:


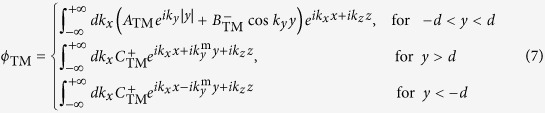



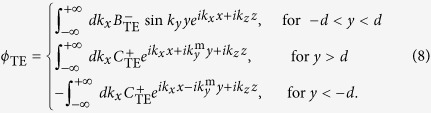


By applying the boundary conditions, we obtain the expressions of the coefficients


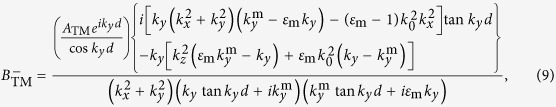



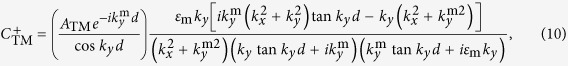



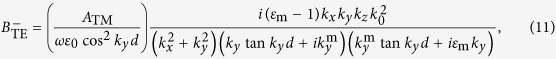



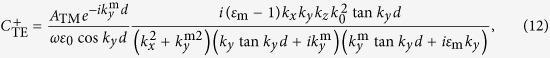


where we have defined 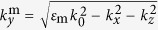. The dispersion relations of the propagating waveguide mode and plasmon modes can be derived by setting 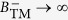,


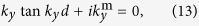



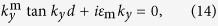



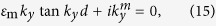


**Figure 2:**
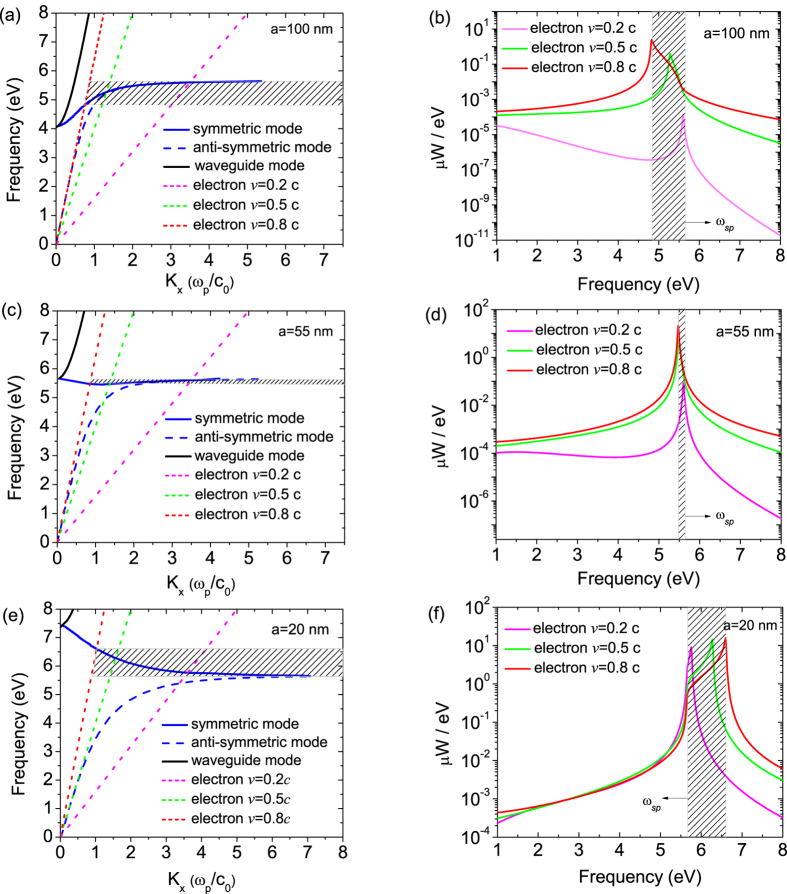
Dispersion curves of the MIM waveguide structure with core thicknesses of *a* = 100 nm (**a**), 55 nm (**c**), and 20 nm (**e**). Dot lines denote the dispersion of an electron in free space at velocities of *v* = 0.2*c*, 0.5*c* and 0.8*c*. Dissipated power spectrum for a moving electron in the MIM structure with core thicknesses of *a* = 100 nm (**b**), 55 nm (**d**) and 20 nm (**f**) at velocities of *v* = 0.2*c*, 0.5*c* and 0.8*c*. Shading region depicts the frequency range for exciting Cherenkov radiation at the electron velocity of *v* = 0.8*c*.

Equations (14) and (15) describe the symmetric and anti-symmetric modes of surface plasmons, respectively. Figure 2(a,c,e) show the dispersion curves of the SPPs in a MIM waveguide structure with core thicknesses of *a* = 100 nm, 55 nm and 20 nm, respectively. The short dashed lines plot the dispersion relation of the electron moving in free space at different velocities and the black solid line plots the dispersion of propagating waveguide mode solved from Eq. (13). The blue solid and dash lines denote the dispersion for symmetric and anti-symmetric surface plasmon mode in the MIM waveguide structure, respectively. In the case we consider where the moving electron is located at the central plane within the waveguide, only the symmetric mode (blue solid line) can be excited. The frequencies at which the Cherenkov radiation can be excited are determined by the intersections between the electron line and the surface plasmon dispersion curve. In the ranges where *k*_*SPP*_ > *k*_*e*_ (shaded region for electron velocity *v* = 0.8*c*), the surface plasmon dispersion relation and the Cherenkov radiation condition are satisfied simultaneously. Thus, the SPPs can be excited and transformed into Cherenkov radiation. For the core thickness of 100 nm as shown in Fig. 2(a), the dispersion branch has a positive slope (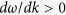), and therefore the group velocity has the same direction with the phase velocity, corresponding to forward Cherenkov radiation. When the core thickness is reduced to 55 nm, the dispersion curve of the symmetric plasmon mode is shifting up and becomes flat with a slope of near zero. The intersection approaches to the asymptotic surface plasmon frequency of *ω*_*sp*_ = 5.65 eV, as shown in Fig. 2(c). As the core thickness is further reduced to 20 nm, the dispersion curve of the anti-symmetric mode is shifting away from that of the symmetric surface plasmon mode. The dispersion branch of symmetric surface plasmon mode shows a distinct negative slope (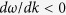). Therefore, the group velocity has the opposite direction to that of phase velocity, and reverse Cherenkov radiation of SPPs occurs at the frequencies below the intersection.

The power dissipated by the moving electron over a time period Δ*t* can be calculated by


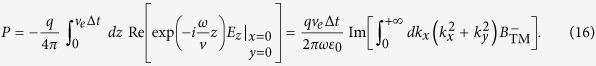


Figure 2(b,d,f) show the dissipated power spectra for a moving electron in the MIM waveguides of different core thickness. We find that the radiation spectrum becomes broader and radiation power becomes higher with the increase of the electron velocity. For the large core thickness (100 nm) case, where only normal Cherenkov radiation can be excited, all the power peaks appear below the surface plasmon frequency, and show a red shift as the electron travels faster. In contrast, for the small core thickness (20 nm) case which allows for reverse Cherenkov radiation, the power peak only appear above the surface plasmon frequency. The increase of the electron velocity results in the blue shift of the power peaks and the increase of the bandwidth. For the critical case (core thickness of 55 nm) with a flat dispersion branch, increasing the electron velocity only yields the rise of the radiation power, while sharp power peaks are observed closely around the surface plasmon frequency irrespective of the electron velocity.

**Figure 3:**
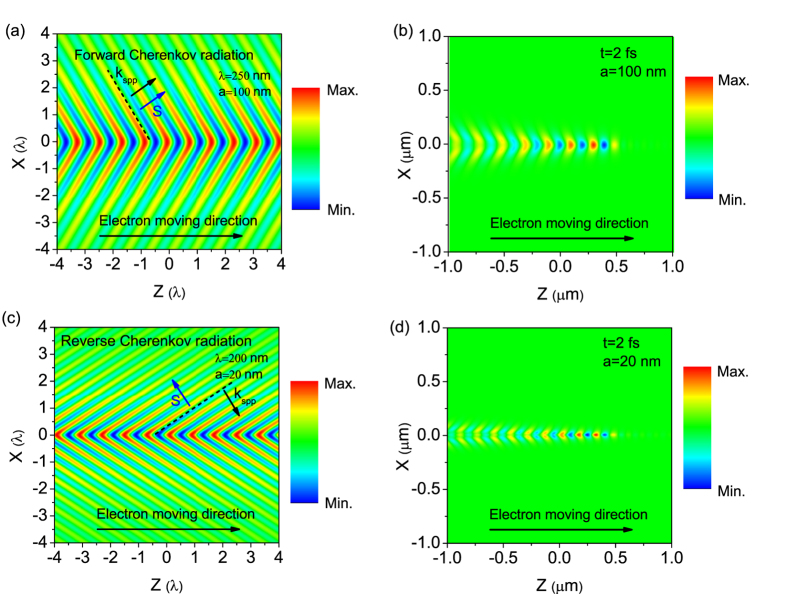
Calculated electric field (*E*_z_) distributions in the MIM structure with core thicknesses of *a* = 100 nm at wavelengths of λ = 250 nm (**a**), and in the MIM structure with *a* = 20 nm at *λ* = 200 nm (**c**), respectively. The *E*_z_ field distribution at 2 *fs* excited by a moving electron in a MIM structure with core thicknesses of *a* = 100 nm (**b**) and 20 nm (**d**), respectively. The electron velocity is *v* = 0.8*c*.

To observe the Cherenkov radiation, we plot the *E*_z_ field distribution at the wavelength of *λ* = 250 nm (4.96 eV) for the MIM waveguide structure with the core thicknesses *a* = 100 nm and the electron velocity of *v* = 0.8*c*, as shown in Fig. 3(a). The electric field distribution in time domain can be calculated by applying Fourier integral of Eq. (6), 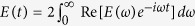, and the electric field distribution at time *t* = 2 *fs* is displayed in Fig. 3(b). We can see that the Poynting power flow is parallel to the surface plasmon wavevector, and the cone of the radiation is pointing forward relative to the motion of the electron. This demonstrates our analysis on the dispersion curves in Fig. 2(a). Figure 2(e) shows the dispersion curves of the MIM waveguide with even a smaller core thickness of 20 nm. Figure 3(c) shows the *E*_z_ field distribution at the wavelength of *λ* = 200 nm (6.2 eV) for the MIM waveguide structure with core thickness of *a* = 20 nm and the electron velocity of *v* = 0.8*c*. As we can see that the direction of Poynting power flow is opposite to that of the surface plasmon wavevector. Figure 3(d) shows the corresponding snapshot of *E*_z_ field distribution at 2 *fs*, where the cone of the radiation is directed reverse to the motion of the electron. (The videos for an electron moving in the MIM waveguide of different core thicknesses at the velocity of 0.8*c* are provided in the Supplemental Material)

**Figure 4:**
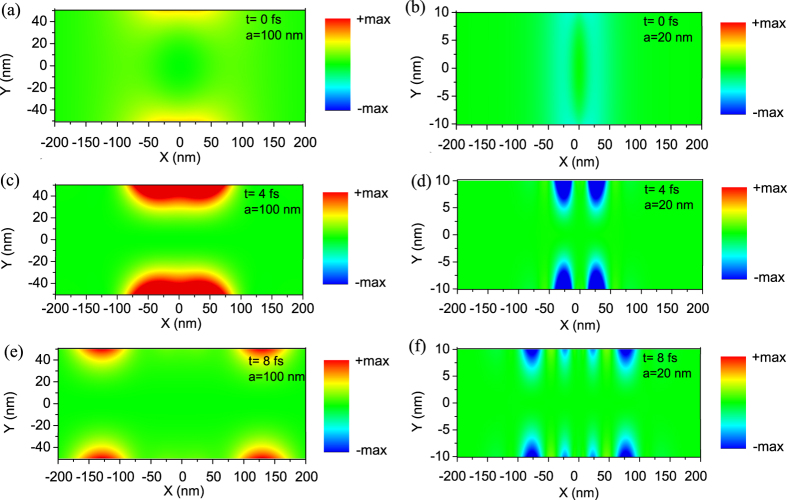
Poynting power flow cross section for an electron moving in a MIM waveguide at different time. (**a**) *t* = 0 *fs*, (**c**) *t* = 4 *fs*, (**e**) *t* = 8 *fs* with waveguide core thickness of *a* = 100 nm. (**b**) *t* = 0 *fs*, (**d**) 4 *fs*, (**f**) *t* = 8 *fs* with waveguide core thickness of *a* = 20 nm. The electron velocity is *v* = 0.8*c*.

To further verify the direction of radiation in different cases, we calculate the Poynting power flow cross section of the MIM waveguide (*x*-*y* plane) at time *t* by 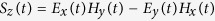. Figure 4 depicts the Poynting power flow cross section at *t* = 0 *fs*, *t* = 4 *fs*, and *t* = 8 *fs*, for the MIM waveguides with core thicknesses of *a* = 100 nm and *a* = 20 nm. The former case shows a positive value while in the latter case, the Poynting power flow cross section is negative in value, corresponding to forward and backward radiated Cherenkov plasmon waves, respectively.

**Figure 5:**
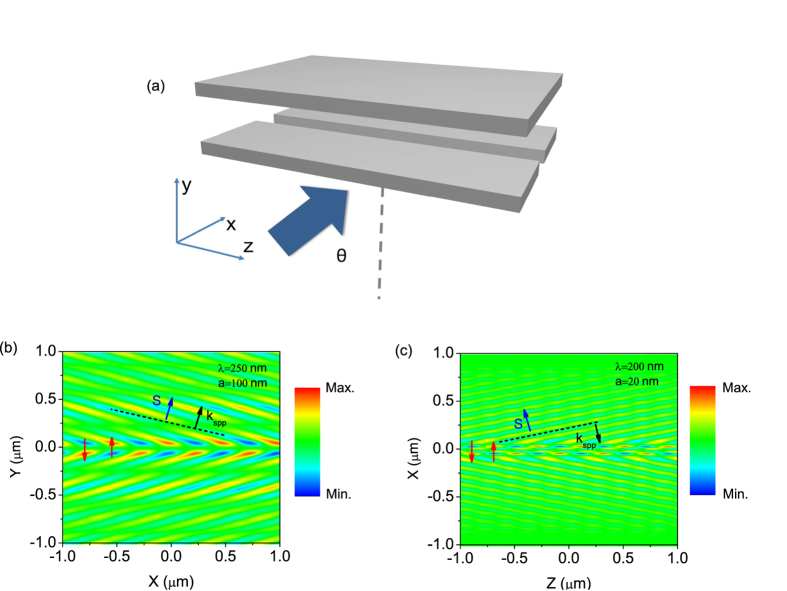
(**a**) Schematic for the excitation of Cherenkov radiation by a slit in the MIM structure with an oblique incident light (incident angle *θ* = 30°). FDTD Simulated electric field (*E*_y_) distributions for MIM structure with core thicknesses of *a* = 100 nm (**b**) and 20 nm (**c**) at wavelengths of 250 nm and 200 nm, respectively.

Finally, we consider a possible practical setup to detect the Cherenkov radiation, and validate our proposal with FDTD simulations. As shown in Fig. 5(a), an S-polarized light (polarization perpendicular to the plane of incidence) is incident on the bottom metal film at an angle *θ*. The excited slit (50 nm wide) generates a running wave of polarization with sinusoidal variable amplitude as analysed by Lee and Genevet^18^,^22^. The moving electron can be modeled by a phase electromagnetic dipole array of an infinite number of elements pointing the direction of the moving electron. This can be treated by the running wave of polarization excited by the slit with a phase profile of 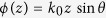 along the slit^15^,^18^. Figure 5(b,c) show the simulated electric field (*E*_y_) distributions in the MIM waveguide structure with core thicknesses of *a* = 100 nm and 20 nm, excited by oblique incident light of *θ* = 30ᵒ at wavelengths of 250 nm and 200 nm, respectively. As agreed very well with our previous analytical investigations, forward and backward Cherenkov radiations can be observed for the two cases.

We highlight that although our calculations (and simulations) are performed for Drude metals, the results obtained also apply to realistic metals. As we have thoroughly show in this paper, the reverse Cherenkov surface radiation only relies on the surface mode with a negative group velocity. Existence of this mode has been confirmed by early experiments^23^. Hence, the relatively large dissipation losses in realistic materials may decrease the amplitude of surface plasmon waves, but do not change the physics reported in our paper.

## Conclusion

We have presented a theoretical approach to investigate the Cherenkov radiation for surface plasmons. Our theory enables us to properly design the plasmonic structures so that reverse Cherenkov radiation can be generated. Since our approach does not require any metamaterial with negative refractive index, it may greatly reduce the difficulty in detecting reverse Cherenkov radiation in experiments. This demonstration of Cherenkov radiation for SPPs is not necessarily restricted to the MIM structures in our discussions, but can be extended to different plasmonic structures in other wavelength ranges and further exploited for other plasmonic materials including 2D materials, such as graphene for infrared range. This theory not only provides an understanding of the properties of Cherenkov radiation in complex plasmonic systems, but also offers great advantages for designing the Cherenkov plasmonic devices, which could be useful for high energy and modern optics applications.

## Additional Information

**How to cite this article**: Tao, J. *et al.* Reverse surface-polariton cherenkov radiation. *Sci. Rep.* **6**, 30704; doi: 10.1038/srep30704 (2016).

## Supplementary Material

Supplementary Information

Supplementary Video 1

Supplementary Video 2

Supplementary Video 3
