# Supplementary material for: Reverse surface-polariton cherenkov radiation
Source: Sci Rep. 2016 Aug 1;6:30704. doi: 10.1038/srep30704 (PMC4967887; doi:10.1038/srep30704)
Supplement: Supplementary Information [file srep30704-s1.pdf]

Supplementary Information for "Reverse surface-polariton cherenkov radiation"

Jin Tao, Qi Jie Wang, Jingjing Zhang, Yu Luo*

School of Electrical and Electronic Engineering, Nanyang Technological University, 639798, Singapore.

*E-mails: luoyu@ntu.edu.sg

Video 1. The time domain field of an electron moving in the MIM waveguide of a core thicknesses *a* = 100 nm.

Video 2. The time domain field of an electron moving in the MIM waveguide of a core thicknesses *a* = 55 nm.

Video 3. The time domain field of an electron moving in the MIM waveguide of a core thicknesses *a* = 20 nm.
